# Cardiomyocyte Formation by Skeletal Muscle-Derived Multi-Myogenic Stem Cells after Transplantation into Infarcted Myocardium

**DOI:** 10.1371/journal.pone.0001789

**Published:** 2008-03-12

**Authors:** Tetsuro Tamaki, Akira Akatsuka, Yoshinori Okada, Yoshiyasu Uchiyama, Kayoko Tono, Mika Wada, Akio Hoshi, Hideki Iwaguro, Hiroto Iwasaki, Akira Oyamada, Takayuki Asahara

**Affiliations:** 1 Muscle Physiology & Cell Biology Unit, Tokai University School of Medicine, Isehara, Kanagawa, Japan; 2 Department of Regenerative Medicine, Division of Basic Clinical Science, Tokai University School of Medicine, Isehara, Kanagawa, Japan; 3 Teaching & Research Support Center, Tokai University School of Medicine, Isehara, Kanagawa, Japan; 4 Department of Orthopedics, Division of Surgery, Tokai University School of Medicine, Isehara, Kanagawa, Japan; 5 Department of Urology, Division of Surgery, Tokai University School of Medicine, Isehara, Kanagawa, Japan; 6 Stem Cell Translational Research, Kobe Institute of Biomedical Research and Innovation/RIKEN Center for Developmental Biology, Kobe, Hyogo, Japan; University of Reading, United Kingdom

## Abstract

**Background:**

Cellular cardiomyoplasty for myocardial infarction has been developed using various cell types. However, complete differentiation and/or trans-differentiation into cardiomyocytes have never occurred in these transplant studies, whereas functional contributions were reported.

**Methods and Results:**

Skeletal muscle interstitium-derived CD34^+^/CD45^−^ (Sk-34) cells were purified from green fluorescent protein transgenic mice by flowcytometory. Cardiac differentiation of Sk-34 cells was examined by *in vitro* clonal culture and co-culture with embryonic cardiomyocytes, and *in vivo* transplantation into a nude rat myocardial infarction (MI) model (left ventricle). Lower relative expression of cardiomyogenic transcription factors, such as GATA-4, Nkx2-5, Isl-1, Mef2 and Hand2, was seen in clonal cell culture. However, vigorous expression of these factors was seen on co-culture with embryonic cardiomyocytes, together with formation of gap-junctions and synchronous contraction following sphere-like colony formation. At 4 weeks after transplantation of freshly isolated Sk-34 cells, donor cells exhibited typical cardiomyocyte structure with formation of gap-junctions, as well as intercalated discs and desmosomes, between donor and recipient and/or donor and donor cells. Fluorescence *in situ* hybridization (FISH) analysis detecting the rat and mouse genomic DNA and immunoelectron microscopy using anti-GFP revealed donor-derived cells. Transplanted Sk-34 cells were incorporated into infarcted portions of recipient muscles and contributed to cardiac reconstitution. Significant improvement in left ventricular function, as evaluated by transthoracic echocardiography and micro-tip conductance catheter, was also observed.

**Conclusions and Significance:**

Skeletal muscle-derived multipotent Sk-34 cells that can give rise to skeletal and smooth muscle cells as reported previously, also give rise to cardiac muscle cells as multi-myogenic stem cells, and thus are a potential source for practical cellular cardiomyoplasty.

## Introduction

**C**ardiac dysfunction induced by myocardial infarction is a leading cause of morbidity and mortality in humans, as injured cardiomyocytes exhibit limited regenerative capacity. Therefore, the notion of cellular cardiomyoplasty, based on transplantation of various cell types including bone marrow stem cells [1], [2], dermal fibroblasts [3], fetal or neonatal cardiomyocytes [4], [5] and skeletal myoblasts [4]–[14], has been proposed, with the expectation that such cells would differentiate and/or trans-differentiate into cardiomyocytes. Among these cell types, skeletal myoblasts have shown numerous advantages, including easy access to donor cells, as autologous myoblasts are readily available from patients without immunosuppression [15]. However, complete trans-differentiation into cardiomyocytes has never occurred [13]. Thus, for best results, i.e., differentiation into cardiomyocytes, autologous adult somatic stem cell transplantation is needed.

We first identified myogenic-vasculogenic progenitor cells in the interstitial spaces of skeletal muscle and purified them by fluorescence-activated cell sorting (FACS) using cluster differentiation cell surface markers (CD34, CD45) after enzymatic isolation [16], [17]. Cells in the CD34^+^/CD45^−^ fraction (Sk-34 cells) formed colonies and had the potential to differentiate into mesodermal cells, such as endothelial cells (ECs), myogenic cells and adipocytes during *in vitro* culture and after *in vivo* transplantation [17]. Sk-34 cells were also confirmed to give rise to ectodermal lineage cells (Schwann cells) after transplantation into severely damaged muscle, with significant functional recovery through the synchronized reconstitution of the muscular, vascular and peripheral nervous systems associated with differentiation into skeletal muscle, vascular smooth muscle, pericytes, endothelial and Schwann cells [18]. These findings suggest that Sk-34 cells are immature stem cells that have epiblastic-like cell capacity, particularly due to their differentiation capacity to mesodermal and ectodermal cell lineages.

During these experiments, we observed that Sk-34 cells spontaneously contracted during cell culture, even in a mononucleated state, in a similar manner to cardiac muscle cells [17] (also present in Movie S1). In addition, Sk-34 cells are able to give rise to skeletal and smooth muscle cells [17], [18]. It is thought that cardiac muscle cells are an intermediate type between skeletal and smooth muscle cells. Thus, our primary hypothesis is that Sk-34 cells can also give rise to cardiac muscle cells (cardiomyocytes) upon receiving differentiation signals from the myocardial micro-environment following co-culture with embryonic cardiomyocytes and/or cell transplantation into cardiac muscle. These cell populations may thus contribute to the functional recovery of damaged heart muscle.

In the present study, we demonstrated that freshly isolated Sk-34 cells can give rise to cardiomyocytes having intercalated discs associated with gap-junctions after transplantation to the MI zone and significantly contribute to functional recovery of the left ventricle. Differentiation into cardiomyocyte was also confirmed by FISH analysis. Consistent with this *in vivo* differentiation capacity, expression of core cardiac network mRNAs (GATA-4, Nkx2-5, isl-1, Mef2c, Hand2 and cardiac muscle actin) associated with skeletal and smooth muscle-specific mRNAs was detected in co-culture with embryonic cardiomyocytes. These findings indicated that Sk-34 cells are multi-myogenic stem cells able to differentiate into cardiomyocytes, in addition to the previously reported differentiation into skeletal and smooth muscle cells, and have implications for therapeutic approaches to treat MI.

## Results

### Differentiation potential of cardiomyocytes *in vitro*

**Figure 1:**
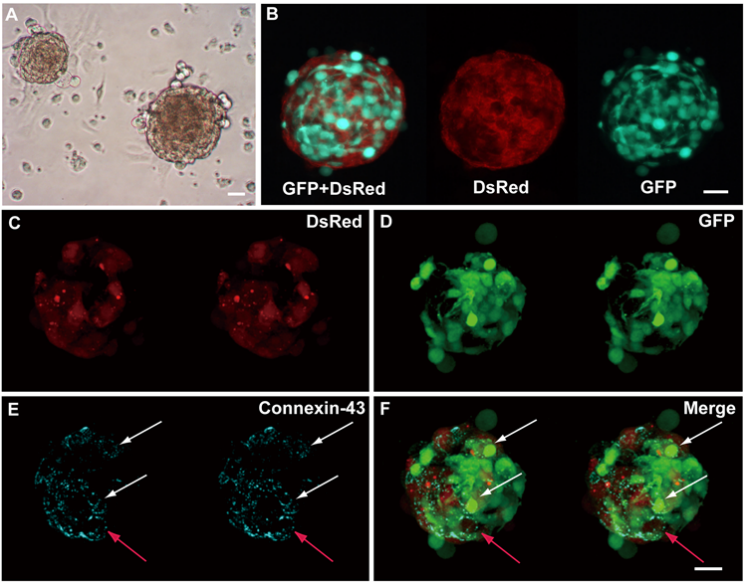
Co-culture of GFP-Tg mouse Sk-34 cells and DsRED-Tg mouse embryonic cardiomyocytes. (A) Typical cardiac spheres in co-culture on glass-slide chamber. (B) Fluorescence microscopic observation of cardiac sphere. Sphere is composed of DsRED^+^ cardiomyocytes and GFP^+^ Sk-34 cells. (C–F) Confocal laser microscopic analysis of cardiac sphere. Dot-like Connexin-43^+^ reactions (gap-junctions) were located around GFP^+^ cells (white arrows in E and F), as well as DsRED^+^ cardiomyocytes (red arrows in E and F). Note that this sphere actively contracted spontaneously and synchronously (video available as Movie S2). There were no yellow (Red+Green) cells suggesting cellular fusion. Scale bars, 20 µm.

Before transplantation, we examined the differentiation potential of Sk-34 cells in normal culture and in co-culture with embryonic cardiomyocytes. In the glass-slide chambers with appropriate cell density, mouse embryonic cardiomyocytes formed sphere-like colonies after 3–5 days of culture (Fig. 1A). The process was as follows: 1) after 1–2 hours in culture, mouse embryonic cardiomyocytes began to plate-down on the glass-slide; 2) synchronous contractions were observed at one day after culture; 3) plate-down cellular confluency increased toward 2–3 days of culture with vigorous synchronous contraction; 4) aggregating cells then began to detach from the glass-slide, probably due to their continuous and rhythmic contractions, and form sphere-like colonies. These sphere-like colonies were not clonal colonies, in contrast to the sphere colonies seen with skeletal muscle-derived stem cells [19, 20, and 21].

Under fluorescence microscopy, there were several GFP^+^ Sk-34-derived cells in the DsRED mouse cardiomyocyte spheres after 6 days of culture (Fig. 1B). These cardiomyocyte spheres showed vigorous spontaneous and synchronous contraction (Movie S2). When these spheres were stained with anti-conexin-43 and observed by confocal laser scanning microscopy (Fig. 1C–F), dot-like staining for gap-junctions was similarly evident around the red cells (red arrows in Fig. 1E and F) and green cells (white arrows in Fig. 1E and F) supporting the synchronous contraction of this sphere (Movie S2). This evidence also suggests that skeletal muscle-derived Sk-34 cells can form gap-junctions with cardiomyocytes and work synchronously (contraction) with host cardiomyocytes *in vitro*, and thus probably differentiate into cardiomyocytes.

**Figure 2:**
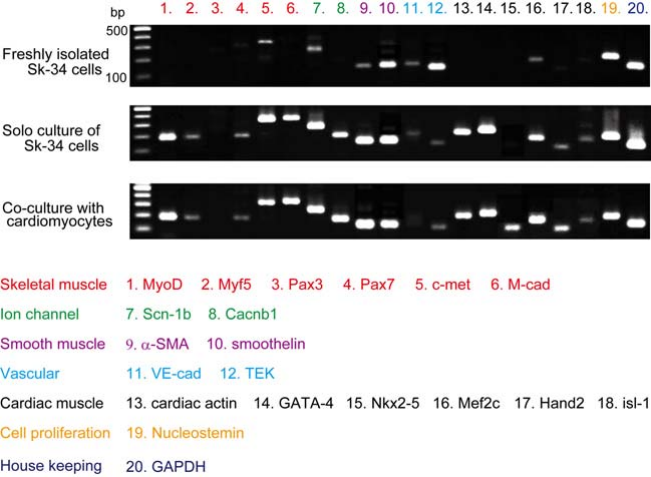
Expression of specific mRNAs for skeletal muscle (1–6), ion channels (7–8), smooth muscle (9–10), vascular markers (11–12), cardiac muscle (13–18) and cell proliferation (19) in freshly isolated cells, and in cells after 5 days of solo culture (without cardiomyocytes) or co-culture with embryonic cardiomyocyte Sk-34 cells. Note that expression of all 6 cardiomyogenic-specific marker mRNAs can be seen after co-culture with cardiomyocytes. M-cad, M-cadherin; Scn1b, sodium channel voltage gated type1-b; Cacnb1, calcium channel voltage-dependent beta-1 subunit; α-SMA, α-smooth muscle actin; VE-cad, VE-cadherin; TEK, tyrosine kinase-endothelial; GAPDH, glyceraldehydes-3-phosphate dehydrogenase.

In order to confirm myocardial differentiation of Sk-34 cells *in vitro*, we performed RT-PCR analysis for GFP^+^ Sk-34 cells after 5 days of co-culture with normal SD rat embryonic cardiomyocytes, as compared with solo culture of Sk-34 cells. After co-culture, all cells were harvested and GFP^+^ cells were sorted by FACS, and mouse skeletal muscle-derived GFP^+^ cells were analyzed by RT-PCR using putative skeletal myogenic, smooth myogenic, ion channel, vascular cell, cardio myogenic, and cell proliferation markers. Freshly isolated Sk-34 cells (before culture) primarily expressed mRNAs for c-met, Scn-1b, α-SMA, smoothelin, VE-cadherin, TEK and Nucleostemin (Fig. 2). Thus, skeletal and cardio myogenic commitment is unlikely, whereas smooth myogenic and vascular commitment are seen. However, both expression of both skeletal myogenic (except for Pax3) and cardio myogenic (except for Nkx2-5) markers can be seen after solo Sk-34 cell culture, and expression of all 6 cardio myogenic markers was seen after co-culture with rat embryonic cardiomyocytes. Interestingly, vascular marker expression decreased after culture, and expression of Nucleostemin was constant, which indicates high cellular proliferation capacity [22].

**Figure 3:**
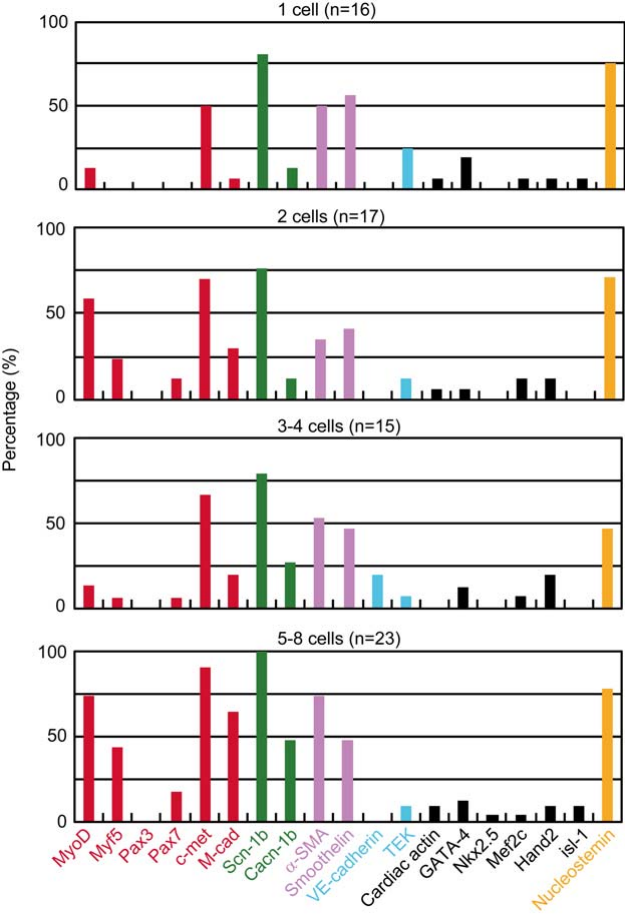
Expression of skeletal muscle (red), ion channel (green), smooth muscle (purple), vascular-related (light-blue), cardiac muscle (black) and cell proliferation (orange) markers before and after cell division of single Sk-34 cells. Cells were cultured clonally in collagen gels. Bar chart shows percentage of samples expressing target mRNA and total numbers are given in parentheses. Percentage of skeletal and smooth muscle, and two ion channel marker-expressing cells gradually increased following cellular divisions. However, cardiac and vascular-related makers did not change during clonal cell culture. M-cad, M-cadherin; Scn1b, sodium channel voltage gated type1-b; Cacnb1, calcium channel voltage-dependent beta-1 subunit; α-SMA, α-smooth muscle actin; TEK, tyrosine kinase-endothelial.

In order to further analyze the cardiomyogenic commitment of Sk-34 cells, we also performed single and 2–8 clonal cells RT-PCR after 3 days of culture (Fig. 3). In this analysis, single cell refers to the stage before cell division, 2 cells refers to the stage after the first cell division, 3–4 cells refers to the second cell division, and 5–8 cells refers to the stage after the third division. Single Sk-34 cells typically showed no expression of Myf5, Pax3, Pax7, VE-cadherin or Nkx2.5, lower % expression of MyoD, M-cadherin, Cacn-1b, TEK, cardiac actin, GATA-4, Mef2c, Hand2 and isl-1, and higher (>50%) expression of c-met, Scn-1b, α-SMA, smoothelin and Nucleostemin mRNAs. This single cell analysis (before cell division) was similar to that for freshly isolated bulk Sk-34 cells, in which elevated markers were positive, reduced markers were sometimes positive or negative, and unexpressed marker were negative (Fig. 2). After the third cellular division, expression of c-met, Scn-1b, α-SMA, smoothelin and Nucleostemin was relatively constant, and MyoD, Myf5, M-cadherin and Cacn-1b gradually appeared after cell division. However, cardiac differentiation-related mRNAs did not dramatically change. These results suggest that cardiac differentiation of Sk-34 cells is not sufficiently induced by clonal cell culture, while skeletal and smooth myogenic differentiation progresses.

Throughout the culture studies, 5 cardiac marker mRNAs (except for Nkx2.5) were expressed during the 5 days of bulk Sk-34 cell solo culture (without cardiomyocytes) (Fig. 2B), and all 6 cardiac markers were sufficiently expressed on co-culture with rat embryonic cardiomyocytes (Fig. 2C), but cardiac differentiation was not induced by clonal cell culture. Thus, it was strongly suggested that cardiac differentiation of Sk-34 cells is accelerated by bulk cell culture and is completely achieved under co-culture. These results also indicated that cell-to-cell relationships, probably including paracrine factors, and cellular milieu are important for stem cell differentiation and development.

### Therapeutic potentials of Sk-34 cells

**Figure 4:**
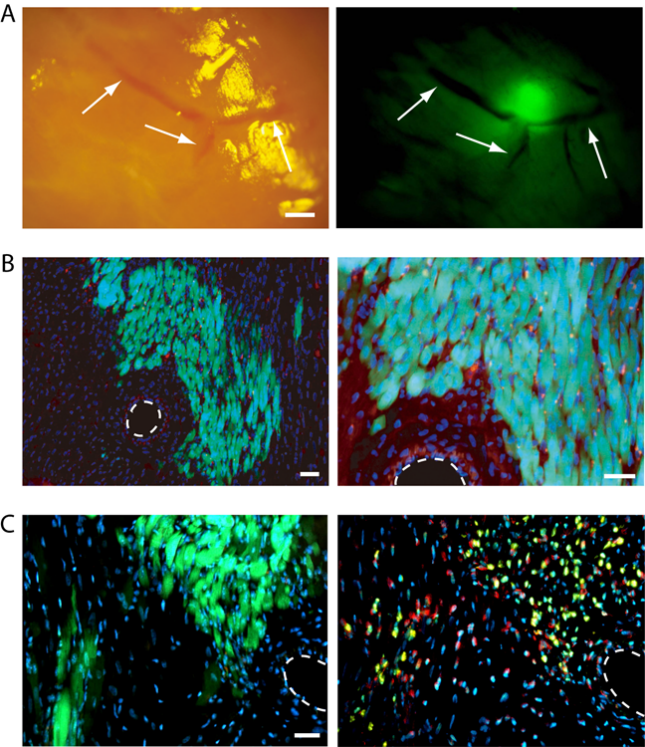
Macroscopic and histological observation and FISH analysis at 4 weeks after therapeutic-bulk cell transplantation. (A) Macroscopic view of implanted GFP^+^ tissues under dissection microscope. GFP^+^ tissues located close to blood vessels (arrows). Scale bar, 500 µm. (B) Histological sections obtained from the region in (A). Left panel, low magnification; right panel, high magnification. Large numbers of GFP^+^ cells are distributed around blood vessels, and cardiac muscle-like cells having apparent striations were seen. Dotted lines indicate blood vessels. Scale bars, 100 µm. (C) FISH analysis for histological serial sections from the region in (B). Distribution of GFP^+^ cells (donor mouse derived) correspond to yellow reactions (mouse genomic DNA probe) among red reactions (host rat genomic DNA probe), thus suggesting that green cells were donor mouse derived. Dotted line indicates blood vessels. Scale bars, 100 µm.

In order to confirm the engraftment and/or therapeutic potential of freshly isolated Sk-34 cells, we performed bulk cell transplantation into a rat MI model. At 4 weeks after transplantation, aggregation of GFP^+^ tissues was seen around blood vessels in the Sk-34 cell-transplanted heart muscle under a dissection microscope (Fig. 4A). On histological sections from corresponding regions, numerous GFP^+^ striated muscle cells similar in appearance to cardiac muscle cells were observed (Fig. 4B). To determine whether these cells were donor derived, we performed FISH analysis using mouse (donor, yellow) and rat (recipient, red) genomic DNA probes (Fig. 4C). On serial sections, distribution of donor GFP^+^ mouse-derived cells clearly corresponded to the distribution of mouse nuclei (yellow), and mixed hybridization of mouse and rat probes (yellow and red) was not seen on the sections. Thus, we concluded that engrafted cardiac muscle-like cells were derived from donor mice, and were not the product of nuclear fusion. Interestingly, the multinucleated myotubes typically seen in skeletal muscle cells and peripheral nerve formations were not detected in the present bulk transplantation study, in contrast to previous transplantation studies in a severe skeletal muscle damage model and into the kidney capsule [17].

**Figure 5:**
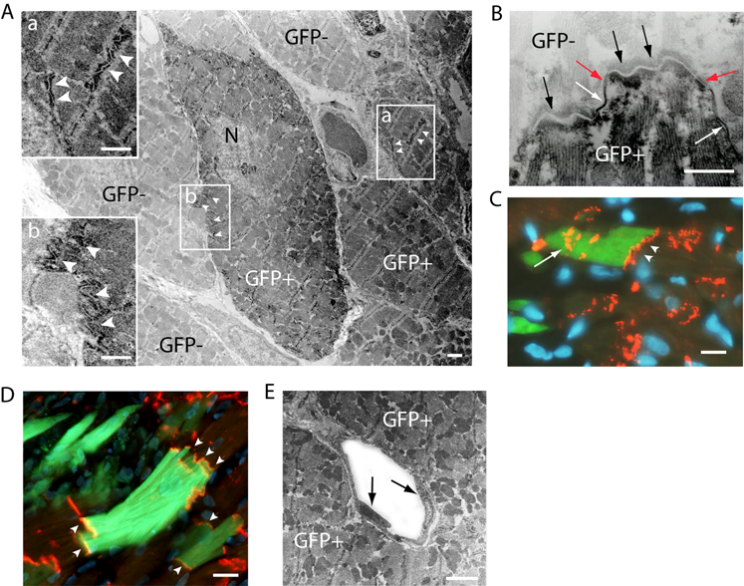
Histological characteristics at 4 weeks after therapeutic-bulk cell transplantation of freshly isolated Sk-34 cells. (A) Immunoelectron microscopic detection of implanted GFP^+^ cells. White squares (a and b) in panel A correspond to the respective insets. Intercalated disks including desmosomes (arrow heads) were clearly evident between GFP^+^ (having dork spots) and GFP^+^ and/or GFP^+^ and GFP^−^ cells. GFP^+^ cells were mononucleated and exhibit typical cardiomyocyte features. N, nuclei. Scale bar, 2 µm. (B) Immunoelectron microscopic detection of gap-junctions and desmosomes (red arrows) between GFP^+^ and GFP^−^ cells (white arrows). Black arrows, fascia adherentes. Scale bar, 2 µm. (C, D) Immunohistochemical detection of gap-junctions between GFP^+^ and GFP^−^ cells (white arrowheads) and GFP^+^ and GFP^+^ cells (arrow) using connexin-43 (red reactions). Scale bar, 10 µm. (E) GFP^+^ endothelial cells in blood vessels were also evident (arrows). Scale bar, 2 µm.

**Figure 6:**
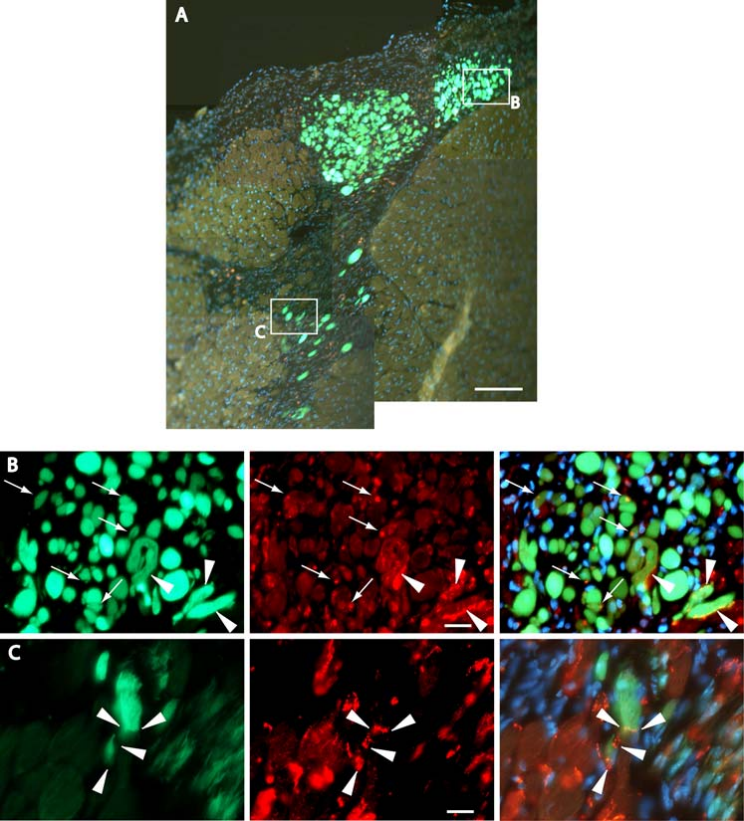
Histological characteristics at 2 weeks after therapeutic-bulk cell transplantation. (A) Contribution to MI zone by transplanted freshly isolated Sk-34 cells. Donor-derived GFP^+^ cells aggregated and filled in the MI zone, even at 2 weeks after transplantation. Squares in panel A correspond to panels B and C. Scale bar, 100 µm. (B and C) Formation of gap-junctions in border (arrowheads) and central (arrows) zones of MI. Elongated connexin-43^+^ reactions (red) were observed in the border zones of MI, while spot reactions were seen in the central zone. Thus, formation of gap-junctions apparently begins in border zones and moves toward the central zone. Scale bars, 10 µm.

We then performed immunoelectron microscopy and immunohistochemical analysis using anti-GFP and connexin-43 antibody in order to further confirm whether engrafted cardiac muscle-like cells were actually cardiac muscle cells (Fig. 5). Engrafted GFP^+ ^(having black dots) cells exhibited typical cardiomyocyte shape with apparent myofilament striations (Fig. 5A). These were mononucleated cells, again suggesting that they were formed independently of cell fusion. In addition, the formation of intercalated disks including desmosomes, a typical characteristic of cardiac muscle cells, was clearly evident between GFP^+^ and GFP^+^ cells (arrowheads in inset a) and/or GFP^-^ and GFP^+^ cells (arrowheads in inset b). Note that typical gap-junctions were also evident between GFP^+^ and GFP^−^ cells (Fig. 5B, white arrows) in addition to desmosomes (B, red arrows). Formation of gap-junctions between GFP^+^ and GFP^−^ cells (Fig. 5C and D, arrowheads) and GFP^+^ and GFP^+^ cells (C, arrow) was also confirmed by connexin43 immunostaining. These results indicate that transplanted Sk-34 cells are incorporated and differentiate into cardiac muscle cells forming desmosomes and gap-junctions under the cardiac muscle milieu. In addition, blood vessels having GFP^+^ endothelial cells were observed (Fig. 5E). Formation of gap-junctions among donor cells and/or between donor cells and recipient cells further suggests that these cells function in the heart as cardiac muscles. In fact, implanted Sk-34 cells already filled in the MI zone at 2 weeks after transplantation (Fig. 6A). In this case, complete formation of gap-junctions was observed only in the border zone of the MI (Fig. 6B and C, arrowheads), and spot-like connexin43^+ ^reactions, probably indicating the early stages of gap-junction formation, were distributed toward the central zone of the MI (Fig. 6B, arrows). Thus, formation of gap-junctions began at the border zones and spread to the central zone of the MI.

### Functional contribution to the MI model

**Figure 7:**
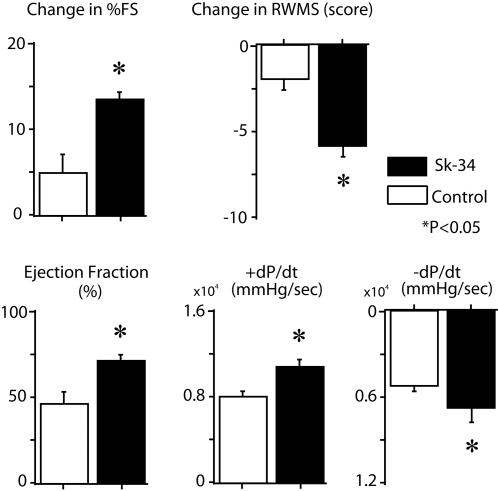
Measurement of LV function after Sk-34 cell transplantation. FS, fractional shortening; RWMS, regional wall motion score; +dP/dt, maximum LV dP/dt; –dP/dt, minimum LV dP/dt. *P<0.05.

The contribution of cell transplantation to cardiac function was examined by functional assessment (see Methods) in freshly isolated Sk-34 cell-transplanted and non-cell-transplanted nude rat MI models. At 4 weeks after transplantation, significantly higher values for all 5 parameters, percentage of fractional shortening (FS), regional wall motion score (RWMS), ejection fraction (%) and maximum and minimum LV dP/dt (+dP/dt and –dP/dt), were observed in the Sk-34 cell-transplanted group when compared with the non-cell-transplanted Control group (Fig. 7). These results strongly suggest a significant contribution to the recovery of LV function, and that transplanted Sk-34 cells differentiate into cardiac muscle in the infarcted myocardium and contribute to maintenance of LV function.

## Discussion

An experimental approach using skeletal myoblast-transplantation for myocardial regeneration was first proposed by Kao et al. [23], and was reported by Mareli et al. [24] as satellite cell transplantation. Subsequently, this “cellular cardiomyoplasty” was established by Chiu et al. [7] Many investigators have since confirmed and expanded on this notion, and it has been reported that improved performance of cryo-injured myocardium occurred in 7/12 rabbits after autologous skeletal myoblast transplantation [14]. Improved functional activity of infarcted hearts transplanted with skeletal myoblasts has since been reported in several studies [8], [10], [12], [14], and thus it is certain that skeletal myoblasts are a practical source of potential cardiomyocytes for myocardial therapy [24]. However, complete trans-differentiation into cardiomyocytes has never occurred in these transplantation experiments, as described in detail by Reineche et al. [13]. Although the formation of gap-junctions and synchronized contraction in skeletal myoblasts has been demonstrated by co-culture with neonatal cardiomyocytes *in vitro* [12], this has never been demonstrated *in vivo* [13]. At 4 or 12 weeks after skeletal muscle stem cell engraftment, cells formed multinucleated, cross-striated myofibers that express fast skeletal myosin heavy chain, but not intercalated disk proteins N-cadherin or connexin-43 [13]. However, the present results for *in vitro* co-culture with embryonic cardiomyocytes and *in vivo* cell transplantation also revealed that skeletal muscle-derived Sk-34 cells are able to differentiate into cardiomyocytes.

The core transcriptional gene networks involved in mammalian cardiac muscle development can be considered as follows; 1) GATA4–Mef2c–Hand2; 2) isl-1–Mef2c–Hand2; 3) Nkx2-5–Mef2c–Hand2 [25]. Interestingly, the present *in vitro* culture data suggest that the former two pathways (1 and 2) can be initiated by solo Sk-34 cell culture, but the third pathway from Nkx2-5 was induced only by co-culture with cardiomyocytes. Further analysis is needed to clarify the expression patterns of these factors in Sk-34 cells, but it is certain that co-culture with embryonic cardiomyocytes induces expression of all three cardiac transcriptional gene networks in Sk-34 cells.

It was recently reported that adult murine skeletal muscle contains cells that can differentiate into beating cardiomyocytes *in vitro*. The paper described cells showing CD34^−^/CD45^−^/C-kit^−^/Sca-1^−^ at initial isolation, and showing round shape, floating and/or weakly attaching behavior, sphere-colony formation and spontaneous beating (contracting) in culture, and that these cells may be different from satellite cells, referring to them as Spoc cells (skeletal-based precursors of cardiomyocytes) [26]. Spontaneous beating (contracting) in culture was similar to the behavior seen in the present Sk-34 cells [17]. However, Sk-34 cells were positive for CD34 and Sca-1, but were whereas negative for CD45 and C-kit. Thus, Spoc cells and Sk-34 cells appear to be different. In addition, the number of CD34-/CD45- cells is small when compared to Sk-34 cells (1∶16∼20) [17], [18], [19] at initial isolation; thus, our method showed 16-20-fold higher efficiency for isolation of potential cardiomyocyte precursors from skeletal muscle over Spoc cells (CD34^−^/CD45^−^/C-kit^−^/Sca-1^−)^. Moreover, our Sk-34 cells are tissue-specific stem cells, not bone marrow derived [17], and are thus useful for direct injection into cardiac muscle, while Spoc cells were injected into blood circulation for cardiac repair [26]. For these reasons, Sk-34 cells can be purified more simply and in much larger quantities, and are more suitable for therapeutic use, as they can be obtained within 3 hours of muscle sampling.

Functional improvement and formation of gap-junctions was observed after transplantation of 7-day cultured satellite cells, but these cells retained the appearance of multinucleated myotubes, while expression of cardiac troponin-T and myosin heavy chain were observed *in vivo* [27]. This further suggests that labeling of cardiomyocyte cytoplasmic phenotype proteins, such as myosin heavy chains, actin and troponins, is not always practical for determining trans-differentiation into cardiac muscle cells in the case of skeletal myoblast transplantation. This is because both cardiac and skeletal muscles are striated muscles, and skeletal muscle myosin heavy chain and troponins are changeable by postnatal physical activity. Evidence, that several antibodies cross-react with both cardiac and skeletal muscle in several species, supports this notion. Therefore, formation of intercalated discs associated with desmosomes and gap-junctions between donor-recipient and/or donor-donor cells, together with typical mononucleated cardiac muscle features, may be practical determinants of cardiac differentiation and/or trans-differentiation in transplanted skeletal muscle-derived stem cells.

To the best of our knowledge, this is the first report demonstrating that skeletal muscle-derived stem cells can give rise to cardiomyocytes associated with the formation of desmosomes and gap-junctions, and incorporating alignment between recipient cardiomyocytes and/or transplanted donor cells (Fig. 5). These results may be reflected the result of significant functional recovery (LV function) after transplantation (Fig. 7). In addition, observed contribution of transplanted Sk-34 cells to the vascular formation (Fig. 5E) may also contribute to functional recovery but their significance is still unclear.

In the present study, cardiac differentiation of Sk-34 cells was confirmed by both *in vitro* co-culture with embryonic cardiomyocytes and *in vivo* cell transplantation (Fig. 1–2 and 5–6). We believe that the presently described cells are able to give rise to cardiac muscle cells for several reasons. First, gentle isolation of the cells appears to be important. Recent findings strongly suggest the importance of cell isolation method, particularly when using enzymatic digestion that lowers proteolytic contamination, in conserving stem cell function in skeletal muscle [28]. We have thus consistently used lower concentrations of collagenase (0.1%) in DMEM when compared with previous studies (1–2%), and we always add 5–10% FBS to the collagenase solution in order to minimize contaminating protease activity and to protect isolated cells. In addition, we did not mince donor muscle before enzymatic digestion (see Methods), which is advantageous for cell isolation with minimum contamination by other cell types, such as committed ECs. In fact, there were no CD31^+^ cells present in the Sk-34 cells [17], in addition to CD133 and NCAM (Figure S1).

For therapeutic use of stem cells, it is also important to maintain cells in an immature state, even after expansion culture. Our previous data shows that the myogenic potential of Sk-34 cells reduces markedly after culture because of undesired differentiation into fibroblast-like cells and/or adipocytes in severely damaged skeletal muscle, and thus Sk-34 cells should be transplanted in a freshly isolated state [18]. The present cell culture and analysis of mRNA expression revealed that cardiac lineage commitment of Sk-34 cells was accelerated association with active commitment to skeletal and smooth myogenic lineages after culture, but multinucleated skeletal muscle cells were scarcely observed after freshly isolated Sk-34 cell transplantation. This result shows differentiation into skeletal myogenic lineage in Sk-34 cells was inhibited after transplantation into cardiac milieu. Thus, early exposure (such as freshly isolated state) to the specific tissue environment apparently leads to preferential commitment and/or differentiation of Sk-34 cells by receiving several damaged tissue-releasing factors.

In addition, Sk-34 cells are considered to be highly immature cells, such as epiblastic-like cells, because of their differentiation into mesodermal and ectodermal cell lineage [18]. Thus, differentiation into cardiomyocytes might occur as a result of milieu-dependent differentiation [29] rather than trans-differentiation. Immature cells receive differentiation signals from their microenvironment, such that the topobiological location of a cell leads to expression of a specific phenotype. Therefore, we also speculate that Sk-34 cells are stem cells remaining in the interstitium of skeletal muscle from the developmental stage. It is likely that these topobiological effects are stronger in the border zones of the MI than that in the central zone, as shown in Figures 4B and C. The therapeutic potential of our stem cells tended to increase when transplanted cells were aggregated in a particular area of the recipient tissue. This suggests the importance of cell-to-cell interactions (paracrine factors) between transplanted cells (see **Differentiation potential of cardiomyocytes** ***in vitro***). These events also contribute to significant functional recovery of the left ventricle (Fig. 7). In fact, when the cells were scattered in the host tissues after transplantation, the therapeutic potential for severely damaged skeletal muscle decreased markedly (data not shown).

The other issue that needs to be addressed is whether our isolated cells are satellite cells, or whether they include satellite cells. This remains uncertain, but the present results are clearly different from those reported for satellite cell transplantation [7], [13], [24], [27]. At present, the most consistent and reliable marker for satellite cells is the paired-box transcription factor Pax7 and formation of satellite cells occurs via the expression of Pax7 and MyoD and/or Myf5 [30], [31], [32]. However, expression of Pax7 mRNA was not detected in freshly isolated and single Sk-34 cells (Figs. 2 and 3). Pax7-independent myogenesis was also clearly demonstrated in Pax7-knockout mice, in which muscle fiber formation occurs despite the absence of satellite cells, although postnatal muscle growth is significantly reduced [30], [31]. Thus, the possible contamination of satellite cells in the Sk-34 cell fraction is extremely low and Sk-34 cells are mainly composed of Pax7-independent myogenic cells.

In conclusion, Sk-34 cells can differentiate into cardiomyocytes with gap-junctions, depending on the cardiac milieu microenvironment, and based on previous reports describing skeletal and smooth muscle differentiation, they are probably multi-myogenic stem cells. This was supported by the evidence from in vitro co-culture with embryonic cardiomyocytes, FISH, fluorescence and immunoelectron microscopical analysis after in vivo cell transplantation. However, the possibility of cell fusion after in vivo transplantation, though small, is apparent and the study would benefit from Cre-loxP system to exclude this possibility. In addition, donor cells also formed blood vessels, as is observed in the case of severe skeletal muscle damage [17]. These results encourage reconsidering the usefulness of autologous cellular cardiomyoplasty using skeletal muscle-derived multi-myogenic stem cells.

## Methods

### Animals

Green fluorescent protein transgenic mice (GFP-Tg mice; C57BL/6 TgN[act EGFP]Osb Y01, provided by Dr. M. Okabe, Osaka University, Osaka, Japan) [33] were used as donor mice in cell transplantation studies. Female athymic nude rats (F344/NJcl-mu/rnu; CLEA, Tokyo, Japan) were used as myocardial infarction (MI) model recipients. GFP-Tg mice were also used for *in vitro* cell culture study with cardiomyocytes from embryos of DsRED-Tg mice (Red mice, C57BL/6pCAGGS-DsRED-1, produced by our laboratory) and normal Sprague-Dawley (SD) rats. All experimental procedures were conducted in accordance with the Japanese Physiological Society Guidelines for the Care and Use of Laboratory Animals as approved by the Tokai University School of Medicine Committee on Animal Care and Use.

### Cell Purification

Whole muscles from the thigh and lower leg (tibialis anterior, extensor digitorum longus, soleus, plantaris, gastrocnemius and quadriceps femoris) of 3-8-week-old GFP-Tg mice were treated with 0.1% collagenase type IA (Sigma-Aldrich) in Dulbecco's modified Eagle's medium (DMEM) containing 5–10% fetal calf serum (FCS) with gentle agitation for 2 h at 37°C and interstitial cells were extracted. Extracted cells were filtered through 70-µm, 40-µm and 20-µm nylon strainers to remove muscle fibers and other debris, and were then washed and resuspended in Iscove's modified Dulbecco's medium (IMDM) containing 10% FCS, yielding enzymatically extracted cells. These cells were stained with biotin-conjugated anti-mouse CD34 (RAM34) and streptavidin-APC, and phycoerythrin (PE)-conjugated anti-mouse CD45 (30-F11). CD34^+^/CD45^−^ (Sk-34) cells were then collected. Live cells were counted after cells positive for propidium iodide (PI) were excluded as dead cells. All antibodies were purchased from Pharmingen (San Diego, CA). Cell analysis and sorting were carried out on a dual-laser FACS Vantage (Becton Dickinson, California, USA).

### Co-culture with embryonic cardiomyocytes

Freshly isolated Sk-34 cells obtained from GFP-Tg mice were cultured with embryonic cardiomyocytes from DsRED-Tg mice (E14-16) and wild-type SD rats (E10-12). Co-culture with mouse cardiomyocytes was used for the “cardiomyocyte sphere formation” experiment and rat cardiomyocytes was used for RT-PCR analysis. Cardiomyocytes were isolated and purified using the “Neonatal Cardiomyocyte Isolation System” (Worthington Biochemical Co., Lakewood, NJ, USA).

For cardiomyocyte sphere formation, isolated mouse cells were applied at 5×10^5^cells/cm^2^ onto glass chamber slides (4 wells; Nalge Nunc International, Tokyo, Japan) and freshly isolated Sk-34 cells were added at 1.5×10^5^ cells/well. Cells were normally cultured in 20%FCS/IMDM for 6 days, and medium was replaced daily. After 6 days of culture, spheres were fixed with 4% paraformaldehyde/0.1 M phosphate buffer (4% PFA/PB), washed with a graded sucrose (0–25%)/0.01 M PBS series and stained using rabbit anti-connexin43 polyclonal antibody (dilution, 1∶1000; incubation, 4°C over-night; SIGMA, Saint Louis, Missouri, USA). Samples were analyzed by light and fluorescence microscopy and confocal laser scanning microscopy (Carl Zeiss LSM-510 META, Jena, Germany).

For RT-PCR analysis, isolated rats embryonic cardiac cells were applied to 2.5×10^6^ cells/3.5 mm culture dishes, and 6×10^5^ Sk-34 cells were added in each dish. Cells were normally cultured in 20%FCS/IMDM for 5 days, after which cultured cells were harvested with trypsin and GFP^+^ mouse skeletal muscle-derived cells were sorted by FACS. GFP^+^ mice Sk-34 cell-derived cells were prepared for bulk cell-specific RT-PCR analysis (see below).

### Single- and clonal-cell RT-PCR

Single-cell RT-PCR was performed based on a highly optimized global RT-PCR procedure [34], [35]. Freshly isolated Sk-34 cells were clonally cultured in collagen-based cell culture (StemCell Tech, see above). After 3 days of culture, single and/or clones composed of 2–8 cells were manually removed with a fine-tip micropipette and suspended/washed in cold RNase-free 0.01M PBS. Samples were then lyzed with 9 µl of cold lysis–first-strand synthesis solution containing first-strand buffer (Invitrogen, Carlsbad, CA), 1% NP-40, 1 mM dithiothreitol, 0.01 mM dNTPs, 3.4 nM dT30-containing primer (AAGCAGTGGTATCAACGCAGAGTGGCCATTACGGCCGTACTTTTTTTTTTTTTTTTTTTTTTTTTTTTTT) and RNase inhibitors (Ambion, Austin, TX; Eppendorf, Hamburg, Germany). Samples were quickly frozen with liquid nitrogen and were stored at −80°C until use. For analysis, samples were heated at 65°C for 5 min and placed on ice. Lysates were equally divided into two PCR tubes, to which 100 U of SuperScript III reverse transcriptase (Invitrogen) or 0.5 µl of nuclease-free water (negative control) was added. The first cDNA strand was synthesized by incubation for 60 min at 45°C. The reaction was stopped by heating at 65°C for 10 min. After cooling on ice, 1.5 µl of 1 U RNase H solution (Invitrogen), 0.5 µl of 75 mM MgCl_2_ and 0.5 µl of nuclease-free water were added to the test sample.

RNA was degraded by incubation for 15 min at 37°C and RNase H was inactivated for 10 min at 65°C. Samples were immediately cooled on ice and 6.5 µl of 2X poly-dA tailing solution containing 2X terminal deoxynucleotidyl transferase buffer, 3 mM CoCl_2_, 1.5 mM dATP and 15 U of terminal deoxynucleotidyl transferase (Promega, Madison, WI) was added to 6.5 µl of first-strand cDNA solution. Poly-dA tailing was performed for 15 min at 37°C and was stopped by heating at 65°C for 10 min. Poly-dA tailed cDNA was pre-amplified using a sequence non-specific two-step PCR protocol with dT30-containing primer. Pre-amplification was performed in a 20-µl volume containing Ex Taq buffer, 1 U of ExTaq-polymerase (Takara Bio, Shiga, Japan), 8.3 µM dT30-containing primer, 0.65 mM dNTPs and 4 µl of poly dA tailed cDNA.

The second strand was synthesized by incubating for 1 min at 94°C, 2 min at 50°C and 2 min at 72°C, followed by 35 cycles of 94°C for 30 s, 60°C for 30 s and 72°C for 2 min. Duplicate reaction tubes were employed in order to compensate for experimental errors. The reaction products from the two tubes were mixed and this mixture was used as a template for the second pre-amplification. The second PCR was performed in a 20-µl volume containing Ex Taq buffer, 1 U of ExTaq-polymerase, 2 µM dT30-containing primer, 0.2 mM dNTPs, and 2 µl of the first PCR product. The reaction comprised 35 cycles of 94°C for 30 s, 60°C for 30 s and 72°C for 2 min.

The second PCR product was subjected to 2% agarose gel electrophoresis and was confirmed as a smear having a peak of 500–700 bp. Specific PCR (30 cycles of 30 s at 94°C, 30 s at 60°C and 2 min at 72°C) was performed in a 15-µl volume containing Ex-Taq buffer, 0.8 U of ExTaq-polymerase, 0.7 µM specific sense and antisense primers, 0.2 mM dNTPs and 0.2 µl of the second PCR product. Preparations without reverse transcriptase served as negative controls in cDNA synthesis, pre-amplification and specific PCR. Amplification of genomic DNA was not detected in specific PCR preparations lacking reverse transcriptase. Specific primers for skeletal, smooth and cardio myogenic stem cells are summarized in Table 1.

**Table 1:** Primers for single cell specific PCR.

Primer	Forward	Reverse
MyoD	GGCCACTCAGGTCTCAGGTGT	TGTTGCACTACACAGCATGCCT
Myf5	CAAGAATGCCTGGTAAATGAAGC	CTGGCTCATGATTGGCAAAG
Pax3	CTTGCTTGAGAACGGGGAAC	CATTTTGGCCAACCTCTGTG
Pax7	AAAAGCACCAAGCCAAGACC	GCACACATCCCACTCACACC
c-met	GCTCGAGAAAGCTGTAATGTGAAAATC	ATTCAGCTCAACTGCAGGTATAGGC
M-cad	GGGCTCTCTCTTGGGATGTG	CTTCTGCACTCTGCCAGGAC
Scn1b	CACATAGGCCACTTCCCACAC	GGGCTAGGAGGTGTCACAGG
Cacnb1	CGTCTCCAACCTCCAGGTACAG	GCTCTTCCCTTCCTTCCTCATG
α-SMA	GCAAACAGGAATACGACGAAGC	GCTTTGGGCAGGAATGATTTG
Smoothelin	CGGCAAGAATGTCTAGCCACTC	TCAAAACGCTGCGTGTGTACA
VE-cadherin	TTGCTGTGTGATAAGCAGTTTGC	TCATGCACCAGGGTGACTAATAGA
TEK	TTCTGCCATGGAGTTACCATCC	AGCAGGTGGCTACCACATCAAC
Cardiac actin	TACCCTGGTATTGCCGATCGT	ACATCTCAGAAGCACTTGCGGT
GATA-4	TGGGACTTTCTCCAGCACAGA	CAATGTTAACGGGTTGTGGAGG
Nkx2-5	TGTCTCGGACCTGGCAGAGC	GGCGACGGCAAGACAACCAG
Mef2c	CACGCCTGTCACCTAACATCC	TGTTAGCTCTCAAACGCCACAC
Hand2	CATTTCTGTCGGGTCGGTTATC	CCACTTAGTTTTAGAGGACGGAAGC
Isl-1	TGTCAGGAGACTTGCCACTTTTC	TCTACATATGGCGCTTTGATTTCAC
Nucleostemin	CGGGCCTGACAAATGGAATAC	ACGACCCGTCAGATGGCTTAC
HPRT	GCAAACTTTGCTTTCCCTGGTTAAG	CAACAAAGTCTGGCCTGTATCCA

M-cad, M-cadherin; Scn1b, sodium channel voltage gated type1-b; Cacnb1, calcium channel voltage-dependent beta-1 subunit; α-SMA, α-smooth muscle actin; VE, vascular endothelial; TEK, tyrosine kinase-endothelial; HPRT, hypoxanthine phosphoribosyltransferase.

In addition, to test the expression of specific markers in freshly isolated and co-cultured GFP^+^ Sk-34 cells with normal SD rat embryonic cardiomyocytes, bulk cell RT-PCR were performed. Freshly isolated and 5 days co-cultured Sk-34 cells sorted as GFP^+^ cells after culture were lyzed and total RNA was purified using a QIAGEN RNeasy micro kit. As a control, 5-day cultured Sk-34 cells under the same conditions of co-culture, except without cardiomyocytes, were also analyzed. First-strand cDNA synthesis was performed with an Invitrogen SuperScript III system using dT30-containing primer (see above), and specific PCR (35 cycles of 30 s at 94°C, 30 s at 60–65°C and 2 min at 72°C) was performed in a 15-µl volume containing Ex-Taq buffer, 0.8 U of ExTaq-HS-polymerase, 0.7 µM specific sense and antisense primers, 0.2 mM dNTPs and 0.5 µl of cDNA. Specific primers for skeletal, smooth and cardio myogenic stem cells for mouse bulk cells are summarized in Table 2.

**Table 2:** Primers for bulk cell-specific PCR.

Primer	Forward	Reverse
MyoD	GGCCACTCAGGTCTCAGGTGT	TGTTGCACTACACAGCATGCCT
Myf5	TTAGCAAACCATGAACACGAAACA	AAGGGGGCTTCATTTACCAGG
Pax3	TGGACAGTCTGCCCACATCTCAGC	GGGAGCCTGTGCTGTAGCAATCAG
Pax7	CCCAACAGGTTTTCCCAACTG	CGGCCTTCTTCTAGGTTCTGCT
c-met	CCAAGCCGCGTATGTCAGTAAA	AAGTCGACGCGCTGCA
M-cad	TGGAGCGTCAGCCAGATTAAC	TTGTCCCGAAGGTCCTCTTG
Scn-1b	CCCTTCTTTTTGCTGATTTGCA	AAAGAGAGGAGGCCAAGAGG
Cacnb1	CGTCTCCAACCTCCAGGTACAG	GCTCTTCCCTTCCTTCCTCATG
α-SMA	GCAAACAGGAATACCGACGAAGC	GCTTTGGGCAGGAATGATTTG
Smoothelin	CGGCAAGAATGTCTAGCCACTC	TCAAAACGCTGCGTGTGTACA
VE-cadherin	TTGCTGTGTGATAAGCAGTTTGC	TCATGCACCAGGGTGACTAATAGA
TEK	TTCTGCCATGGAGTTACCATCC	AGCAGGTGGCTACCACATCAAC
cardiac actin	TACCCTGGTATTGCCGATCGT	ACATCTCAGAAGCACTTGCGGT
GATA-4	GGCGGGACAGTCATGATAGCAG	GAGGGAGAAACAGCGAAAATG
Nkx2-5	CGGAACGACTCCCACCTTTAG	TGGGATGGATCGGAGAAAGG
Mef2c	CACGCCTGTCACCTAACATCC	TGTTAGCTCTCAAACGCCACAC
Hand2	CATTTCTGTCGGGTCGGTTATC	CCACTTAGTTTTAGAGGACGGAAGC
isl-1	TGTCAGGAGACTTGCCACTTTTC	TCTACATATGGCGCTTTGATTTCAC
Nucleostemin	CGGGCCTGACAAATGGAATAC	ACGACCCGTCAGATGGCTTAC
GAPDH	CATCCTGCACCACCAACTGC	ACGCCACAGCTTTCCAGAGG

M-cad, M-cadherin; Scn1b, sodium channel voltage gated type1-b; Cacnb1, calcium channel voltage-dependent beta-1 subunit; α-SMA, α-smooth muscle actin; VE, vascular endothelial; TEK, tyrosine kinase-endothelial; GAPDH, glyceraldehydes-3-phosphate dehydrogenase.

### Myocardial Infarction Model and Bulk Cell Transplantation

In an effort to confirm the engraftment and/or therapeutic potential, we performed bulk cell transplantation into a myocardial infarction (MI) model. MI was induced in female nude rats under halothane anesthesia (Fluothane, Takeda Chemical, Osaka, Japan). After tracheal insertion and initiation of ventilation (room air, rate 60 cycles/min, tidal volume 1 ml/100 g body weight, Harvard Apparatus Rodent Ventricular, model 683), the heart was exposed by left thoracotomy. The proximal left coronary artery was ligated and/or blocked-off with an electrical knife. Freshly isolated Sk-34 cells were prepared from GFP-Tg mice and transplanted into the infarcted LV wall of female nude rats. Cells (5–8×10^5^) were suspended in 5–10 µl of DMEM, then injected slowly into the damaged portion (discolored patch) using a fine tip glass pipette. These therapeutic bulk cell transplantations were performed in 5 rats and were prepared for morphological analysis.

For functional assessment, rats were anesthetized with ketamine and xylazine (IP, 60 mg/kg and 10 mg/kg, respectively), and MI was induced by ligating the left anterior descending coronary artery. Twenty minutes after MI, rats received intramyocardial transplantation of Sk-34 cells suspended in 10 µl of DMEM (n = 8) or the same volume of DMEM without cells (control group, n = 8). After injection was completed, rats were allowed to recover.

### Immunostaining and Immunoelectron Microscopy

Transplanted hearts were perfused and fixed overnight with 4% paraformaldehyde/0.1 M phosphate buffer (4% PFA/PB), washed with a graded sucrose (0–25%)/0.01 M PBS series, and quick-frozen in isopentane, and several 7-µm cross-sections were then obtained. Localization of gap-junctions was detected using rabbit anti-connexin43 polyclonal antibody (dilution, 1∶1000; incubation, 4°C; overnight; SIGMA, Saint Louis, Missouri, USA). Reactions were visualized using Alexa Fluor-594-conjugated goat anti-rabbit antibodies (1∶500, room temperature, 2 h; Molecular Probes, Oregon, USA). For immunoelectron microscopy, sections were stained using rabbit anti-GFP antibody (1∶300, 4°C overnight; Molecular Probes) and HRP-conjugated anti-rabbit antibody (1∶200, 4°C overnight; Dako, Carpinteria, California, USA). Reactions were visualized with DAB after fixation in 1% glutaraldehyde/0.1 M phosphate buffer. Sections were then fixed in 1% osmium tetroxide/0.05 M phosphate buffer and were prepared for electron microscopic analysis.

### Fluorescence *in situ* Hybridization (FISH) Analysis

In order to confirm the intrinsic plasticity (not nuclear fusion) of Sk-34 cells, we performed FISH analysis. For this purpose, genomic DNA was extracted from nude rat and C57BL/6 mouse livers and genomic DNA probes were labeled with biotin-dUTP or digoxygenin-dUTP by nick translation. Sections were washed with PBS and treated with 0.5% pepsin/0.1 M HCl for 90 s at 37°C, and were then washed again and dehydrated in 70% and 100% ethanol at room temperature for 2 min each. Genomic DNA probes were then applied and denatured at 90°C for 13 min, and hybridized overnight at 37°C in a humidified chamber. Post-hybridized sections were stringency washed with 50% formamide/2x SSC and 1x SSC. Hybridization was visualized using anti-digoxygenin-Cy3 (red reactions, rat chromosomes) and streptavidin-FITC+anti-FITC-Alexa594 (yellow reactions, mouse chromosomes). Nuclei were counterstained with DAPI.

### Physiological Assessment of LV Function Using Echocardiography and Micro-tip Conductance Catheter

Transthoracic echocardiography (SONOS 5500, Philips Medical Systems) was performed to evaluate LV function at 4 weeks after MI and cell transplantation. Freshly isolated Sk-34 cells (n = 8) and non-transplanted Controls (DMEM only group, n = 8) were prepared for functional analysis. Under general anesthesia with ketamine and xylazine, LV end-diastolic and end-systolic dimensions (LVEDD and LVESD, respectively) and fractional shortening (FS) were measured at the mid-papillary muscle level. Regional wall motion score (RWMS) was evaluated as per published criteria. Immediately after the final echocardiography, the rats underwent cardiac catheterization for more invasive and precise assessment of global LV function, as described previously. A 2.0 Fr micro manometer-tipped conductance catheter (SPR 838; Millar Instruments Inc, Houston, TX) was inserted via the right carotid artery into the LV cavity. LV pressure and its derivative (LV dP/dt) were continuously monitored using a multiple recording system (Pressure-Volume Conductance System ARIA and Pressure-Volume Analysis Using P-V Analysis Software [Millar Instruments Inc, Houston, TX] and Power Lab® DAQ System [AD Instruments, Australia]). LV ejection fraction (EF) and the maximum and minimum LV dP/dt (+dP/dt and –dP/dt, respectively) were continuously recorded for 20 min. All data were acquired under stable hemodynamic conditions.

## Supporting Information

Figure S1 FACS analysis for CD133 and NCAM (neural cell adhesion molecule)-positive cells in enzymatically extracted cells from mouse skeletal muscle, and a comparison with Sk-34 cells. Cells were obtained from GFP-Tg mouse muscles similarly as transplanted and/or cultured cells. In fractionated cells, CD133-positive cells were completely CD45 positive and Sk-34 cells were CD45 negative; thus, CD133-positive cells did not include Sk-34 cells. In addition, there were no NCAM-positive cells among the cells enzymatically extracted from mouse skeletal muscle.
(1.58 MB TIF)

Movie S1 Spontaneous contraction of Sk-34 cells after 7 days of culture. Sk-34 cells were initially cultured in liquid IMDM with 20% FBS for 2 days (2×104/35-mm dish). Medium was then replaced with 0.5% methylcellulose containing 5% FBS/IMDM without cytokines. Contracting cells were typically floating and/or weakly attached.
(3.43 MB MOV)

Movie S2 Spontaneous and synchronous contraction of cardiomyocyte sphere obtained by co-culture of DsRED-Tg mouse embryonic cardiomyocytes and GFP-Tg mouse Sk-34 cells. Enzymatically isolated cardiomyocytes were cultured on glass-slide chambers (5×105cells/cm2 densities) with 1.5×105 cells/well Sk-34 cells in 20%FCS/IMDM for 6 days. Medium was changed for everyday. This synchronously contracting sphere corresponds to the sphere analyzed in Figure 1.
(3.11 MB MOV)
