# Supplementary figures and images for: Cardiomyocyte Formation by Skeletal Muscle-Derived Multi-Myogenic Stem Cells after Transplantation into Infarcted Myocardium
Source: PLoS One. 2008 Mar 12;3(3):e1789. doi: 10.1371/journal.pone.0001789 (PMC2262151; doi:10.1371/journal.pone.0001789)

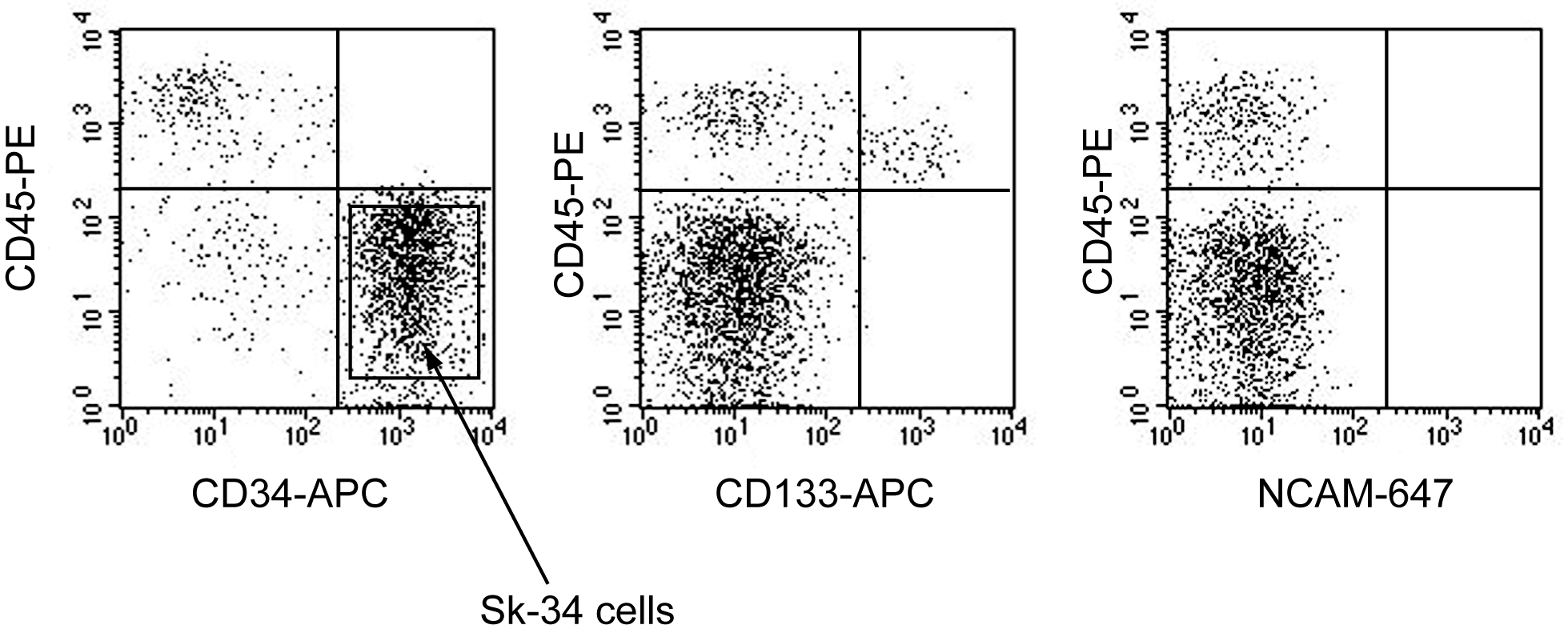

Supplement: Figure S1 — FACS analysis for CD133 and NCAM (neural cell adhesion molecule)-positive cells in enzymatically extracted cells from mouse skeletal muscle, and a comparison with Sk-34 cells. Cells were obtained from GFP-Tg mouse muscles similarly as transplanted and/or cultured cells. In fractionated cells, CD133-positive cells were completely CD45 positive and Sk-34 cells were CD45 negative; thus, CD133-positive cells did not include Sk-34 cells. In addition, there were no NCAM-positive cells among the cells enzymatically extracted from mouse skeletal muscle. (1.58 MB TIF) [file pone.0001789.s001.tif]
